# Multivalent and Bidirectional Binding of Transcriptional Transactivation Domains to the MED25 Coactivator

**DOI:** 10.3390/biom10091205

**Published:** 2020-08-19

**Authors:** Heather M. Jeffery, Robert O. J. Weinzierl

**Affiliations:** 1 Department of Life Sciences, Imperial College London, London SW7 2AZ, UK; heather.jeffery@path.ox.ac.uk; 2 Sir William Dunn School of Pathology, University of Oxford, Oxford OX1 3RE, UK

**Keywords:** transactivation domain, coactivator, mediator, MED25, VP16, ETV5, ERM, ‘fuzzy’ complex, intrinsically disordered, molecular dynamics simulation, bidirectional binding, computational prediction

## Abstract

The human mediator subunit MED25 acts as a coactivator that binds the transcriptional activation domains (TADs) present in various cellular and viral gene-specific transcription factors. Previous studies, including on NMR measurements and site-directed mutagenesis, have only yielded low-resolution models that are difficult to refine further by experimental means. Here, we apply computational molecular dynamics simulations to study the interactions of two different TADs from the human transcription factor ETV5 (ERM) and herpes virus VP16-H1 with MED25. Like other well-studied coactivator-TAD complexes, the interactions of these intrinsically disordered domains with the coactivator surface are temporary and highly dynamic (‘fuzzy’). Due to the fact that the MED25 TAD-binding region is organized as an elongated cleft, we specifically asked whether these TADs are capable of binding in either orientation and how this could be achieved structurally and energetically. The binding of both the ETV5 and VP16-TADs in either orientation appears to be possible but occurs in a conformationally distinct manner and utilizes different sets of hydrophobic residues present in the TADs to drive the interactions. We propose that MED25 and at least a subset of human TADs specifically evolved a redundant set of molecular interaction patterns to allow binding to particular coactivators without major prior spatial constraints.

## 1. Introduction

The regulated expression of the human genome results from the interplay between epigenetic processes and the activity of gene-specific transcription factors (GSTFs) [1]. Specific parts of GSTFs, the transactivation domains (TADs) [2] are responsible for stimulating the expression of nearby genes by interacting dynamically with coactivators that are typically part of the invariant basal transcriptional machinery. Some of the best understood coactivators are present in the basal factor TFIID and in the Mediator complex that associate with RNA polymerase II during transcription initiation [3,4]. TADs activate transcription in a gene-specific manner by aiding in the recruitment and/or subsequent stabilization of the basal transcriptional machinery at promoters. Although discovered more than three decades ago, the structural and functional basis of coactivator-TAD interactions is still poorly understood. This is mostly because these interactions are relatively weak (micro-/high nanomolar affinities), short-lived (characterized by high association and dissociation rate constants) and structurally highly dynamic [5,6,7,8]. Research on one of the best-understood model systems, the interaction between the yeast transcription factor GCN4 and its coactivator target GAL11, has demonstrated that these interactions are “fuzzy”, which means that they are best understood as a stochastic equilibrium of rapidly interconverting structures [7,9]. Computational simulation techniques, especially atomistic molecular dynamics (MD) simulations, are therefore ideally suited to provide new insights into such complex interaction patterns [9].

**Figure 1:**
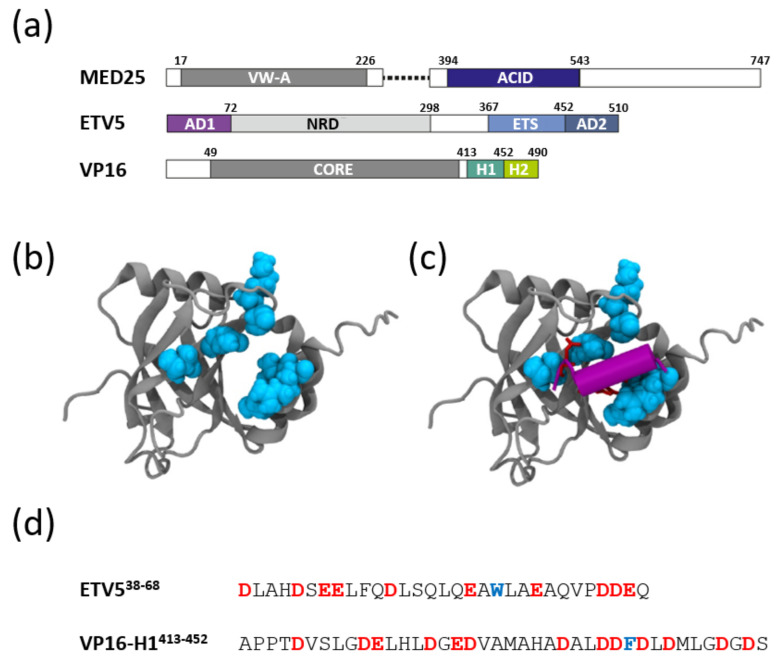
MED25-ACID domain interactions with transactivation domains (TADs). (**a**) Domain structure of MED25, ETV5 and VP16. Various functionally characterized domains and their location within the primary structures are shown as colored boxes. The numbers indicate amino acid positions at the border. MED25: VW-A (von Willebrands factor domain A; dark grey); ACID (activator interacting domain; dark blue). ETV5: AD1, AD2 (transactivation domain 1 (purple) and 2 (pastel blue), respectively); NRD (negative regulatory domain; light grey); ETS (ETS DNA-binding domain, powder blue). VP16: Core (Oct-1/HCF-binding domain; dark grey); H1 and H2 (transactivation domains H1 (turquoise) and H2 (lime green), respectively). (**b**) The solution structure of the MED25-ACID domain (residues 394–543) is shown as a grey cartoon structure. Note the seven-stranded β-barrel structure flanked by three α-helices. Residues identified as contributing to the binding of the VP16-H1 TAD (K^413^ and P^414^ (in the loop joining β1 and β2 sheet); I^449^ and Q^451^ in β3; V^534^, I^537^ and R^538^ in α3) are shown in cyan with their van der Waals radii. (**c**) Same as in (**b**), but with the ETV5^50–61^ TAD modelled as a purple cartoon structure in the position and orientation proposed previously [16]. Some of the key sidechains (L^53^, W^57^ and E^60^) are modelled as liquorice structures and face the TAD-binding region as defined by the MED25 residues listed previously. (**d**) Primary amino acid sequence of the ETV5^38–68^ and the VP16-H1^413–452^ TADs. Negatively charged residues are highlighted in red and large hydrophobic amino acids shown to be functionally particularly relevant in blue.

Here, we apply a computational approach to investigate the interactions of a human and a viral TAD with the activator-interacting domain (ACID) of the Mediator subunit MED25 (Figure 1a). MED25 has recently emerged as one of the most significant targets for functional interactions with a range of transcriptional activators [4,6,8,10,11,12,13,14,15].

The structure of the human MED25 Activator Interaction Domain (“ACID”) consists of a seven-stranded closed β-barrel with three externally located α-helices [17] (Figure 1b). Site-directed mutagenesis studies identified a hydrophobic pocket that plays a critical role for binding the TAD of the VP16 protein encoded by Herpes virus, as well as the TADs of cellular transcription factors, such as the ‘Ets-related molecule’ (ETV5; also known as ERM; (Figure 1a–c)). ETV5 has been implicated in controlling aspects of cell differentiation and proliferation, immune response, apoptosis and cancer [18]. One of the TADs of ETV5 (AD1; Figure 1a) has been narrowed down to a short motif located near the N-terminus of the protein (ETV5^38–68^; Supplementary Materials Figure 1) [19]. This sequence is intrinsically disordered but binds in an α-helical conformation to the MED25-ACID of the mediator complex (Figure 1c,d) [16,20]. The VP16-TAD is substantially longer and consists of two separate parts, referred to as subdomains H1^413–452^ and H2^453–490^ (Figure 1a) [21]. NMR titration experiments revealed that the two subdomains bind on opposite faces of MED25-ACID and that the VP16-H1^413–452^ TAD binds in a region overlapping with the ETV5 binding site [12,13,16]. The interactions of the TADs of ETV5^38–68^ and VP16-H1^413–452^ with MED25-ACID are thus arguably some of the experimentally best-defined examples of human TAD-coactivator interactions, yet the exact binding mechanism remains largely unknown. While we expect to observe similarities in the binding behavior of these two TADs, we were also interested to find out whether there are variabilities in the observable binding behaviors that may reveal differences in their functional properties. ETV5 is expected to cooperate in an orderly fashion with other GSTFs in human cells to regulate the expression of around 200 genes [22,23], whereas the viral VP16-TAD has primarily evolved to disrupt transcription of the host cell by competing with existing TAD-coactivator complexes [24]. Furthermore, other highly studied coactivators contain TAD-binding sites that are comparatively ‘flat’ and allow the transactivating helix of TADs to bind varying up to 180^o^ in spatial orientation according to computational simulations [16]. In contrast, the TAD-binding region of MED25 is much more cleft-like and thus likely to accommodate significantly less rotational freedom in its fuzzy binding mode (Figure 1c) [12,13]. We were therefore particularly interested to investigate how the ETV5 and VP16 TADs respond to binding to such a spatially restricted target region.

## 2. Materials and Methods

### 2.1. Modelling, Parameterization and MD Simulation

The coordinates of ETV5^50–61^ docked to MED25 (*cis*-orientation) published in Landrieu et al. [16] were kindly provided to us by A. Verger. The portion of ETV5^50–61^ present in the model was extended N- and C-terminally to the longer ETV5^38–68^ TAD based on the primary sequence from UniProtKB-P41161 (www.uniprot.org) to match it more precisely to the portion of the protein used in the experimental work [16]. The extensions were performed in Yasara Structure [25] using various helical φ and ψ parameters to prevent steric clashes of the added residues with the coactivator surface (due to the flexibility of TADs, the precise position of residues in the starting model is not directly relevant because it will equilibrate during the subsequent simulation steps). For creating the *trans*-orientation model, the TAD was rotated by 180^o^ but otherwise kept in the same position relative to MED25. The resulting models were capped at N- and C-termini (acetyl- and amide groups, respectively) to neutralize their charges, parameterized with Amber ff14SB [26] and set up in a TIP3P water box extending 25 Å from the protein surface and containing 150 mM NaCl [27]. For creating models of VP16-H1^413–452^ bound to MED25, in both *cis*- and *trans*-orientations, residues in the ETV5^50–61^/MED25 model were mutagenized in silico to the appropriate primary amino acid sequence based on UniProtKB-P06492. A 5000-step steepest descent minimization and 5000-step conjugated gradient minimization with a non-bonded cut-off of 10 Å and a positional restraint of 500 kcal/mol/Å^2^ were included in the first minimization step. The second minimization step involved a 1000-step steepest descent minimization and 1500-step conjugated gradient minimization with a non-bonded cut-off of 10 Å without any constraints. Following the minimization, the system was heated up to 310K linearly by a 100-ns simulation with 10,000 steps and positional restraints of 10 kcal/mol/Å^2^. A standard simulation was then performed for 30–40 nanoseconds at 310K with 2 femtosecond time steps, 10 Å cut-off and no constraints on GPUs [28] to obtain equilibrated values for dihedral- and total potential energy using AMBER16 [29]. These values were used to set up conditions for one microsecond-long accelerated (torsion and potential energy dual boost) molecular dynamics (aMD) simulations with an α-factor of 0.2 in a constant-temperature, constant-pressure (NPT) ensemble as previously described [9,30].

### 2.2. Markov Chain Monte Carlo (MCMC) Simulations

The Phaistos program package for protein structure inference was used to create Markov chain Monte Carlo (MCMC) simulations of individual TADs [31,32]. For both ETV5^38–68^ and VP16- H1^413–452^, two independent sets of MCMC simulations were set up with 25 threads each. To avoid any structural bias from starting structures, the simulations were started using the amino acid sequence as the sole input. The pivot-uniform backbone and uniform sidechains moves create a random, uniformly distributed rotation of the dihedral (φ, ψ) and sidechain torsion angles (χ angles) in single residues. The energy terms were integrated with the Profasi force field that is parameterized to simulate interactions in the presence of a solvent. The Metropolis-Hastings algorithm was used as the acceptance criterion.

### 2.3. Analysis and Visualization

The trajectory data were analyzed by various methods provided by Visual Molecular Dynamics (VMD; [33]), Yasara Structure (preparing structures for simulation) [25] and CPPTRAJ (molecular distance and angle measurement, secondary structure quantitation) [34]. Relative angles between MED25 and TADs were measured using vectors representing the directions of β3 (MED25^447–455^ [17]) and α-helical portions of ETV5 (ETV5^45–59^) or VP16 (VP16^436–447^).

### 2.4. MM-GBSA Analysis

The binding free energy was estimated by the Molecular Mechanics Generalized Born Surface Area (MM-GBSA) method [35]. From each aMD simulation, snapshots were extracted at 1 nanosecond intervals to sample the whole course of the trajectory.

## 3. Results

### 3.1. Structural Aspects of ETV5^38–68^ and VP16-H1^413–452^ TADs Prior to Binding to Coactivators

**Figure 2:**
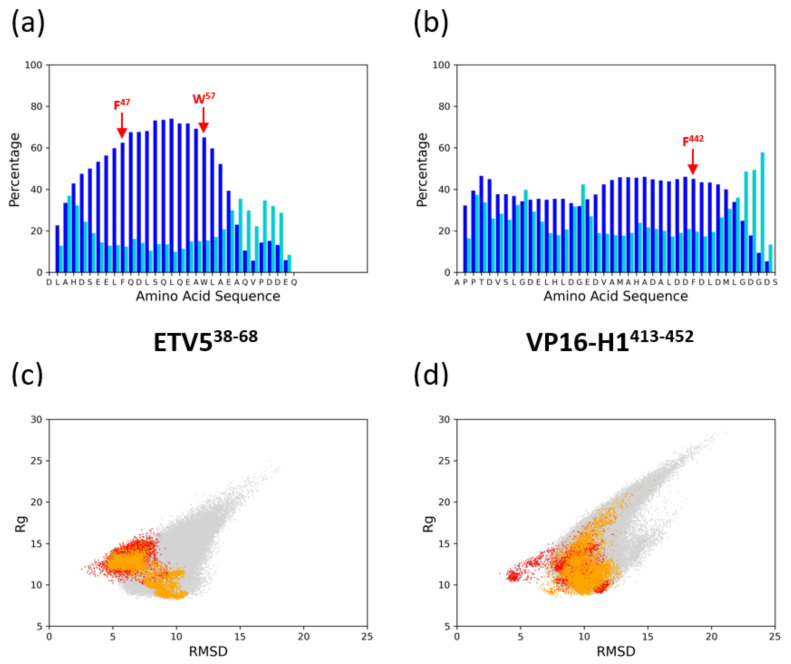
Secondary structure propensities of transactivation domains (TADs) ETV5^38–68^ and VP16-H1^413–452^. (**a**) Markov chain Monte Carlo (MCMC) simulation of the ETV5^38–68^ TAD. The percentage of helical (blue) and ‘bend/turn’ (turquoise) conformations formed in the simulated structures is plotted as a bar chart relative to the primary amino acid sequence (horizontal axis; single letter amino acid code; residues shown start at position 36 and end at position 68 relative to the full length sequence). (**b**) Same as in (**a**), but for the VP16-H1 primary amino acid sequence. The horizontal axes are residues 413 to 452 of the full-length sequence of VP16 shown as a single-letter amino acid code. (**c**) Phase diagram plotting the root-mean square deviation (RMSD) against the radius of gyration (R_g_) for the MCMC-simulated data (light grey), and the MED25-bound versions of ETV5^38–68^ in the *cis* (red) and *trans* (orange) orientation (see text for more details). (**d**) Same as in (**c**) but for the VP16-H1^413–452^ TAD.

TADs are intrinsically disordered but generally display a propensity for the formation of transient α-helices [9,19,36]. In order to explore the full innate secondary structure potential of ETV5^38–68^ and VP16-H1^413–452^ TADs in the absence of any coactivator binding, we carried out Markov chain Monte Carlo (MCMC) simulations that allow for the comprehensive exploration of conformational states without entrapment in local minima (Figure 2a,b).

In close agreement with experimental data, the MCMC data predicts a α-helicity for the unbound ETV5^38–68^ TAD (~39% experimental [19] versus 44% simulated; Table S1). Much of the helicity is observed in the central region containing large hydrophobic amino acids (L^46^, F^47^ and W^57^). The VP16-H1^413–452^ TAD also adopts a high α-helical content including—in contrast to the ETV5^38–68^ TAD—a significant proportion of 3_10_ helices (Table S1). Overall, the ETV5^38–68^ TAD appears to contain more highly localized secondary structures with higher helical propensity as compared to VP16-H1^413–452^. A feature common to both TADs is a rapid increase in disorder at the C-terminus, where the last five to six residues are increasingly adopting ‘bend’ and ‘turn’ conformations (Figure 2a,b). Both TADs also include distinct motifs in their primary sequences that are characteristic of transcriptional activators (Figure S1) [2,37,38].

### 3.2. The ETV5^38–68^ and VP16-H1^413–452^ TADs Interact with MED25 in an Orientation-Specific Manner

**Figure 3:**
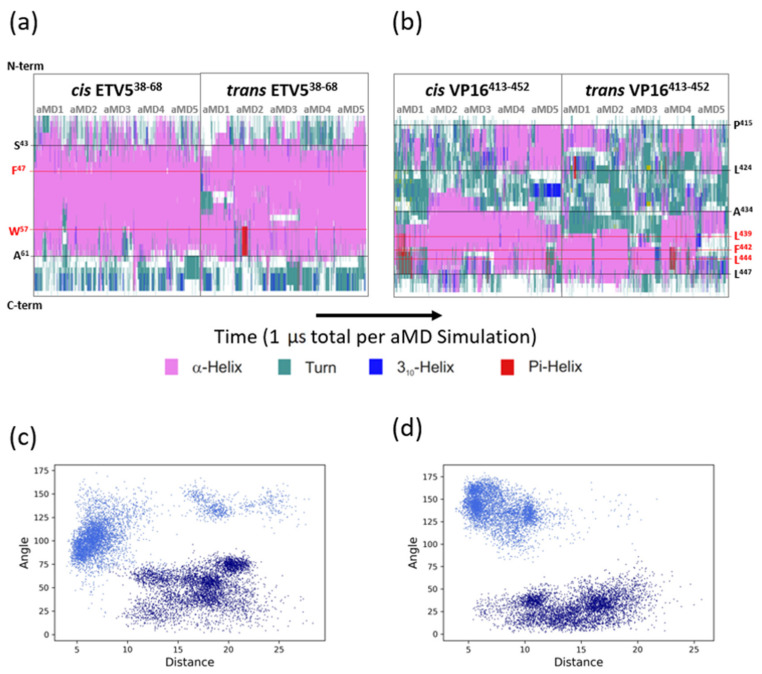
Time-course of TAD structures bound to MED25-ACID. (**a**) Secondary structure assignments along the vertical primary amino acid sequence are shown across the five independent accelerated molecular dynamics (MD) simulations (aMD1-aMD5) for the *cis*- and *trans*-orientations of ETV5^38–68^ relative to MED25. The secondary structure state of each residue is color-coded according to the color scale shown below. The positions of two key amino acid residues, F^47^ and W^57^, are highlighted in red and with a dotted line across all ten ETV5^38–68^ aMD trajectories. The positions of residues S^34^ and A^61^, marking the approximate N- and C-terminal limit of the α-helical region, respectively, are shown with a black dotted line. (**b**) Same as in (**a**), but for the VP16-H1^413–452^ TAD data. The position of three key amino acid residues, L^439^, F^442^ and L^444^, are highlighted in red and with a dotted line across all ten VP16-H1^413–452^ aMD trajectories. The positions of residues P^415^ and L^424^, marking the approximate N- and C-terminal limit of the most N-terminal α-helical region, respectively, are shown with a black dotted line. For the C-terminal helical region, positions of A^434^ and L^447^, demarcating the approximate boundaries of the C-terminal helix, are shown. (**c**) Phase diagram showing the distance of ETV5-W^35^ relative to MED25-Q^451^ across all *cis-* (light blue) and *trans*- (dark blue) simulations. Note that in the *cis*-orientation ETV5-W^35^ typically remains closely associated (typically with 5–7 Å) with the cleft on MED25 (of which MED25-Q^451^ is an integral part). In the *trans*-orientation, ETV5-W^35^ constantly remains distant (typically 10–22 Å) from the cleft. (**d**) Same as in (**c**) but for the distance of VP16-F^422^ relative to MED25-Q^451^. See Figure S2 for a more detailed break-down of the angle distributions between MED25 and the TADs in each of the four simulations.

Experimental observations, including in vitro competition data, support the idea that the ETV5^38–68^ and VP16-H1^413–452^ TADs bind to the same target area on MED25-ACID [16]. We were therefore interested in finding out to what extent the binding mode of these two distinct TADs share common features or differ from each other. In the absence of further structural information, we modelled the VP16-H1^413–452^ TAD in the same starting position as ETV5^38–68^. Moreover, because we do not know in which orientation the ETV5^38–68^ and VP16-H1^413–452^ TAD bind to MED25, we created two models containing each of the TADs in the two possible orientations, which we will refer to as either the *cis*- or *trans*-orientation, respectively. For all models, we assumed that the initial position, secondary structure content and local structure would only be of minimal significance because the TADs would adopt an appropriate position and structure during the course of the aMD simulations (‘induced fit’; [13]). In order to sample a diverse range of conformations, the MED25-ACID/ETV5^38–68^ and MED25-ACID/VP16-H1^413–452^ models were subjected to five independent aMD simulations from the same starting conformation lasting for 1 microsecond each (Figure 3). Due to the nature of the enhanced sampling simulation strategy, the trajectories are expected to reveal motions that occur in a time range spanning several hundred microseconds, or even into the millisecond range, thus bringing the simulations into a physiologically relevant time-range that is directly comparable to the durations of interactions typically observed in vitro and in vivo (millisecond range) [5,7,8,16]. We started our simulations from a model with an essentially arbitrary secondary structure outside the predicted α-helical cores spanning residue positions 50–61 of ETV5. Any specific secondary structures formed in this region therefore represent structures formed by de novo folding on the coactivator surface. The secondary structure analysis shows that the C-terminal border of α-helical structure of ETV5 coincides mostly with A^61^ as proposed by the previously available model (Figure 3a) [16]; occasional transitions of helical structures to less ordered ones are temporary and appear readily reversible.

In contrast, at the N-terminal end, we observe a variable extension of α-helical structures that routinely extend to S^43^ and, less frequently, beyond. The borders of α-helical conformation appear to form independently in both *cis*- and *trans*-orientations in comparable locations, suggesting that the different structural features of the coactivator surface encountered in these opposite orientations of the TAD play no substantial role in affecting the formation of secondary structures - the borders of the α-helix are independently encoded within the primary structure of the ETV5-TAD itself (Figure 2a). While all five simulations of ETV5 in the *cis*-orientation give rise to uninterrupted α-helices, two simulations of ETV5 in the *trans*-orientation (aMD1 and aMD3) result in localized internal breaks of helicity that occur shortly after the start of the simulation and persist until the end, suggesting that they are stable, alternative conformations of ETV5^38–68^ binding to MED25. The α-helices observed during the course of the aMDs (even when disrupted as in some of the *trans*-orientation ETV5 simulations) ensure that key hydrophobic residues (such as F^47^ and W^57^), that are known to play an essential role in coactivator binding [16,20], remain almost constantly embedded within a stable local helical TAD conformation, regardless of TAD orientation relative to the coactivator. The overall conformations explored when bound to the coactivator surface fall within the conformational space that ETV5^38–68^ explores as a free polypeptide (Figure 2c). While binding in the *cis*-orientation creates a spatially rather confined structure (red data points in Figure 2c), binding in the *trans*-orientation bifurcates phase space into two distinct states that appear to be populated comparably (orange data points in Figure 2c). We conclude that the binding of ETV5^38–68^ in both *cis*- and *trans*-orientations has only minor effects on secondary structure, but that binding in the *trans*-orientation at least partially relies on accessing conformations that are specific to that orientation (but nevertheless already preconfigured in the unbound TAD).

A similar analysis of secondary structure formation of the VP16-H1^413–452^ TAD reveals a different picture: the α-helices formed when bound to MED25 are shorter and their boundaries are less clearly defined. Two separate regions with α-helical propensity are apparent spanning approximately residues 415 to 424 (the ‘N-terminal helix’) and residues 434 to 447 (the ‘C-terminal helix’; Figure 3b). The N-terminal border of the N-terminal helix is clearly defined due to the presence of two proline residues (P^414^ and P^415^). In two simulations of the VP16-H1^413–452^ TAD in the *cis*-orientation (aMD4 and aMD5), the helical structure appears quite stable once formed, whereas, in the three other simulations, helical structures form only fleetingly. In the *trans*-orientation, characteristic helices with C-terminal borders around S^419^ or L^420^ make an appearance. Previous mutagenesis work has not revealed any residues of special functional significance in this region [21,39]. The C-terminal helix, which includes the functionally significant residues L^439^, F^442^ and L^444^, is partially α-helical in both *cis-* and *trans*-orientation, but with enhanced helicity in the *cis*-direction. Similar to ETV5^38–68^, the key hydrophobic residues are embedded within regions of high α-helical propensity (Figure 3b). The overall structure of the VP16-H1^413–452^ TAD bound to MED25 lies mostly within the conformational space predicted by the MCMC simulation data of the unbound TAD but binding in the *cis*-orientation does appear to draw the TAD into a slightly different conformational space (Figure 2d). This suggests that the surface of MED25 causes a slight deviation from the inherent structural propensity of VP16-H1^413–452^ due to local interactions. Interestingly, the division of the VP16-H1^413–452^ TAD into two separate α-helical parts was unknowingly anticipated from mutagenesis experiments carried out by Cress and Triezenberg (1991) when they showed that substitutions of A^432^ and A^436^ with proline have no detectable functional consequences [39]. While the authors interpreted this as an indication that the whole VP16-H1 TAD was not α-helical, our computational simulation data now show that there is a subregion that is never significantly engaged in forming such a structure.

### 3.3. Energetic Aspects of Bidirectional Interactions of ETV5^38–68^ and VP16-H1^413–452^—TADs with MED25

**Figure 4:**
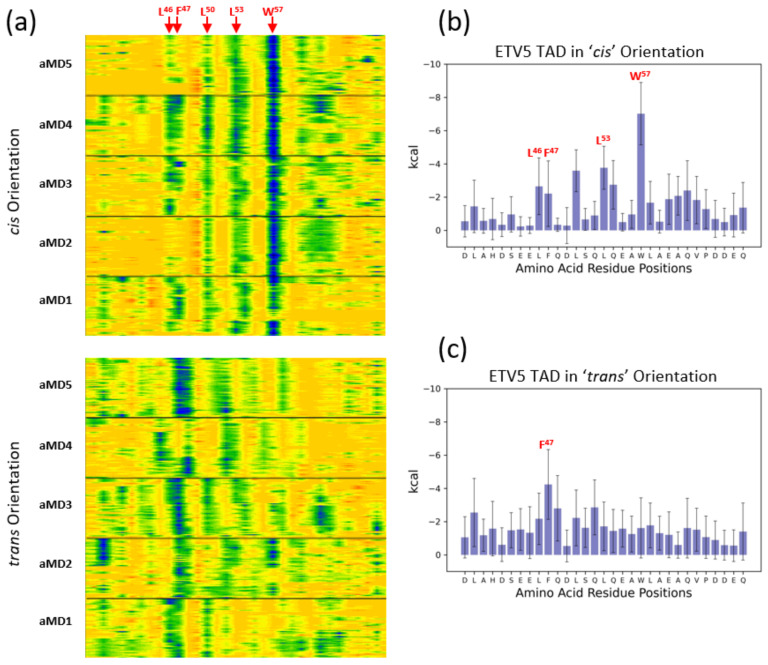
Molecular Mechanics Generalized Born Surface Area (MM-GBSA) analysis of the ETV5 complex with MED25. (**a**) Contour map of the van der Waal’s contribution of TAD residues to binding MED25 in either the *cis-* (top) or *trans*-orientation (bottom) over the time course of five independent aMD simulations (aMD1–5) each. The amino acid sequence is represented from left to right, with the positions of some key amino acids shown by arrows on top. Dark blue colors highlight the strongest contributions to binding. The y-axis represents simulation time (1 µs/simulation). (**b**) Bar chart of the data presented in the top diagram of panel (**a**), quantitating the van der Waal’s contributions of each amino acid in the ETV5^38–68^ TAD on a per-residue basis in the *cis*-orientation. The values shown are the average across all five aMD simulations sampled at 1 nanosecond intervals, the error bars indicate the standard deviations of the measurements (5000 samples). (**c**) Bar chart of the data presented in the bottom diagram of panel (**a**), quantitating the van der Waal’s contributions of each amino acid in the ETV5^38–68^ TAD on a per-residue basis in the *trans*-orientation. The values shown are the average across all five aMD simulations sampled at 1 nanosecond intervals, the error bars indicate the standard deviations of the measurements (5000 samples).

Measurements of the angles of the TAD relative to the coactivator and distances of various key hydrophobic residues provide a further analytic method for visualizing any potential variability in the binding mode. As expected from a comparable model system in yeast [7,9], ETV5^38–68^ binds to MED25 in a variety of angles that typically vary up to 60^o^ within individual simulations and over a wider range in independent simulations (Figure S2). A phase space diagram combining the angle of the TADs with the distance data for ETV5-W^57^ or VP16-F^442^ relative to MED25-Q^451^ shows little, if any, structural overlap between the *cis* and *trans* binding modes (Figure 3c). A Molecular Mechanics Generalized Born Surface Area (MM-GBSA) analysis of intramolecular forces mediating the binding of the ETV5^38–68^ TAD to MED25 in both orientations reveals further details regarding orientation-specific van der Waals interactions (Figure 4). In the *cis*-orientation, ETV5-W^57^ is clearly the major and most constant contributor, while certain residues located more N-terminally (L^46^, F^45^, L^50^, and L^53^) only make occasional and/or minor energetic contributions to ΔG_Binding_. In the *trans*-orientation, we observed a high variability in the distances of ETV5-W^57^ relative to a set of hydrophobic/charged residues (K^413^, P^414^, I^449^ and Q^451^) present on the surface of MED25 (Figure 1b,c); the van der Waals contributions from W^57^ become essentially negligible in such a situation, and ETV5-L^46^, F^47^ and Q^48^ take over a more dominant role aided by multiple additional contacts spread out throughout the TAD (Figure 4a,c).

**Figure 5:**
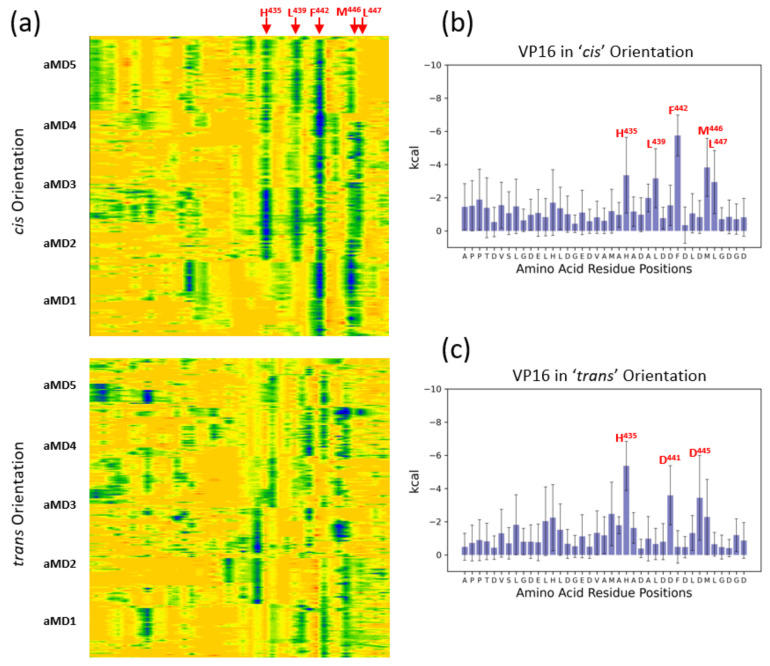
Molecular Mechanics Generalized Born Surface Area (MM-GBSA) analysis of the VP16-H1 complex with MED25. (**a**) Contour map of the van der Waal’s contribution of TAD residues to binding MED25 in either the *cis-* (top) or *trans*-orientation (bottom) over the time course of five independent aMD simulations (aMD1–5) each. The amino acid sequence is represented from left to right, with the positions of some key amino acids shown by arrows on top. Dark blue colors highlight the strongest contributions to binding. The y-axis represents simulation time (1 µs/simulation). (**b**) Bar chart of the data presented in the top diagram of panel (**a**), quantitating the van der Waal’s contributions of each amino acid in the VP16-H1^413–452^ TAD on a per-residue basis in the *cis*-orientation. The values shown are the average across all five aMD simulations sampled at 1 nanosecond intervals, the error bars indicate the standard deviations of the measurements (5000 samples). (**c**) Bar chart of the data presented in the bottom diagram of panel (**a**), quantitating the van der Waal’s contributions of each amino acid in the VP16-H1^413–452^ TAD on a per-residue basis in the *trans*-orientation. The values shown are the average across all five aMD simulations sampled at 1 nanosecond intervals, the error bars indicate the standard deviations of the measurements (5000 samples).

MM-GBSA analysis of the VP16-H1^413–452^ TAD showed the contribution of several residues to the binding event with MED25. Binding in either the *cis*- or *trans*-orientation involves around four to five dominant residues each, but only VP16-H^435^ is shared between both sets (Figure 5; note that some of the residues shown (H^435^, D^441^ and D^445^ are electrostatically charged, but these data sets only take their van der Waals contributions into account)).

We conclude that the ETV5^38–68^ and VP16-H1^413–452^ TADs bind to MED25 in the two experimentally identified *cis-* and *trans*-orientations with conformational differences utilizing different large hydrophobic residues for energetically stabilizing their interactions with MED25. The two separate and partially non-overlapping modes are demonstrated by phase-diagrams (Figure 2c), as well as MM-GBSA analyses (Figure 4 and Figure 5). Interestingly, MM-GBSA analyses of the total ΔG_Binding_ energies predict no substantial differences in binding strengths between the different orientations of either TAD (Figure S3).

### 3.4. MED25 Coactivator Surfaces Interacting with ETV5- and VP16-H1 TADs

**Figure 6:**
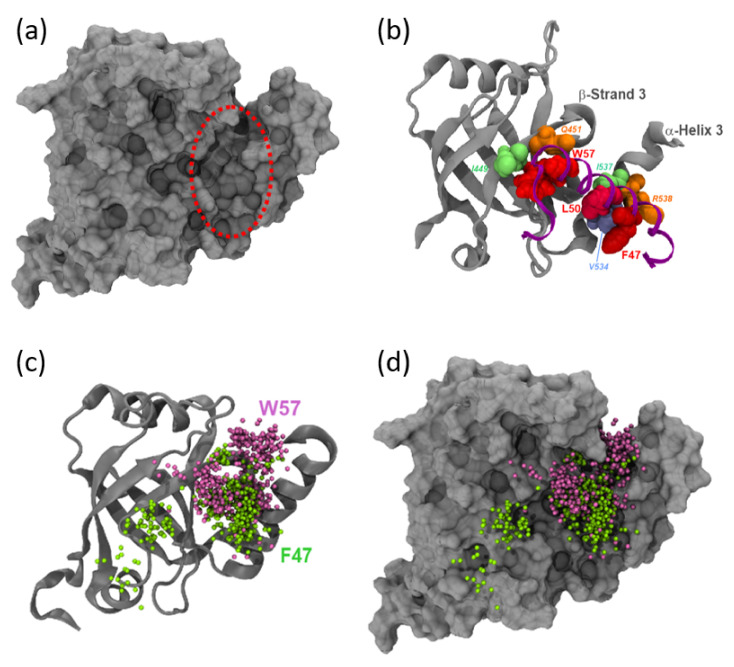
Binding of the ETV5^38–68^ TAD to the MED25-ACID surface. (**a**) Surface view of MED25-ACID. A large binding pocket (red dashed circle) formed at the interface between the β-barrel and α-helix 3 is evident (compare with panel (**b**) for the positions of these secondary structure elements). The molecule is shown in the same orientation in all subsequent panels. (**b**) Representative snapshot of the ETV5^38–68^ TAD in *cis*-orientation (after 200 ns of aMD simulation; *cis* ETV5-aMD1). Three large hydrophobic side chains in the ETV5^38–68^-TAD (F^47^, L^50^ and W^57^) are shown as van der Waals space-filling models in various shades of red. Similarly, key interaction residues within MED25 are shown in space-filling representation and labelled in italics: β3 strand I^449^ (lime green), Q^451^ (orange); α-3 helix V^534^ (light blue), I^537^ (lime green) and R^538^ (orange). (**c**) The superimposed positions of C_α_-atoms of residue F^47^ (green spheres) of ETV5^38–68^ in the *cis*- and W^57^ (purple spheres) of ETV5^38–68^ in the *trans* configuration are shown at 10 nanoseconds (aMD-simulation time) intervals; spatial clusters reveal the preferred location of these atoms at different stages of the simulations. (**d**) Same as in (**c**), but MED25 shown in surface mode to emphasize presence of various clefts and pockets.

We next investigated the position of the ETV5-TAD relative to landmarks present on MED25. Visualization of the MED25 surface reveals a “cleft” that could serve as a binding site for the key hydrophobic residues within the ETV5-TAD (Figure 1b,c; Figure 6a). Available NMR data for MED25-ACID bound to ETV5^38–68^ TAD and VP16-H1^413–452^ has highlighted several residues (MED25 I^449^ and Q^451^ in β3; V^534^, I^537^ and R^538^ in α3) as being involved in TAD binding interactions [16].

Inspection of the trajectories with ETV5 in the *cis*-orientation reveals further insights into the results obtained from the MM-GBSA analysis described earlier. In the most common conformations, ETV5-W^57^ occupies a pocket formed by the surface of the β-barrel structure of MED25. At the same time, the more N-terminally located hydrophobic residues (L^46^, F^47^, L^50^ and L^53^) identified previously as making occasional and/or additional energetic contributions to ΔG_Binding_ (Figure 4) interact predominantly with a cluster of hydrophobic residues located in the nearby α-helix H3 of MED25 (nomenclature based on Bontems et al. [17]). The bipartite binding pattern anchors the TAD through strong hydrophobic forces in two separate positions and thus results in a considerable stabilization of the angle of the ETV5-TAD relative to the coactivator surface (Figure 2b).

With the ETV5-TAD binding in the *trans* position, F^47^ occupies a similar position within the β-barrel pocket as compared to W^57^ in the *cis*-orientation (Figure 6c,d). Under these conditions, there is no significant additional binding contribution by W^57^ (Figure 4 and 5a), accounting for the much more variable angle of the TAD relative to the coactivator surface (Figure 2b). A critical role for F47 has been proposed previously [16]. The substitution of F^47^ with leucine reduces the transactivation potential considerably, and substitution with proline essentially abolishes the activity of the TAD, both in yeast and human cells [19]. These results therefore reflect the functional consequences of substitutions in key residues of TADs interacting with Mediator [40].

**Figure 7:**
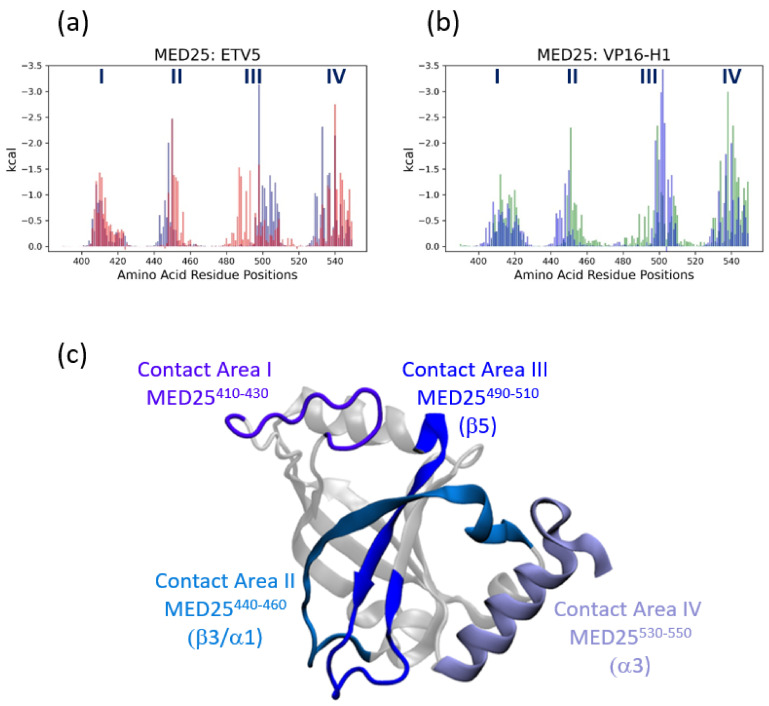
MED25 regions involved in binding ETV5 and VP16-H1 TADs. (**a**) Result of MM-GBSA analysis to highlight MED25 residues making van der Waals contributions to binding of ETV5^38–68^. The bars identifying MED25 residues involved in binding ETV5^38–68^ in the *cis*-orientation are shown in blue, and the bars identifying MED25 residues involved in binding ETV5^38–68^ in the *trans*-orientation are shown in red. The residue numbers shown along the X-axis show the positions within the full-length MED25 protein [17]. The roman numerals on top identify four distinct contact areas (I-IV). See Figure S4 for more details. (**b**) Result of MM-GBSA analysis to highlight MED25 residues making van der Waals contributions to binding of VP16-H1^413–452^. The bars identifying MED25 residues involved in binding VP16-H1^413–452^ in the *cis*-orientation are shown in blue, and the bars identifying MED25 residues involved in binding VP16-H1^413–452^ in the *trans*-orientation are shown in green. (**c**) The MED25 ACID structure—as used in the simulations—with regions shown to contact either ETV5^38–68^ or VP16-H1^413–452^ TADs in *cis*- and *trans*-orientations highlighted in different shades of blue. The identities of specific secondary structure elements present in contact regions II, III and IV are based on the nomenclature from [17].

The binding positions of F^47^ and W^57^ within the MED25 cleft take up slightly distinct areas within the cleft, but also overlap partially (Figure 6c; Figure 7a). This cleft thus constitutes the major binding sites for different bulky hydrophobic residues present in TADs. Our data also show that, depending on the orientation, either ETV5-F^47^ or W^57^ anchor the TAD to these sites. Our observations are in general agreement with conclusions from coupled cluster single(s) and double(s) excitation measurements (CCSD) that showed that the ETV5^38–68^ TAD form a rapidly interconverting conformation ensemble [16]. The simulation data specifically suggest that the biophysical results are most likely based on an equilibrium between *cis* and *trans* binding modes with corresponding emphasis on the differing roles for W^57^ or F^47^, respectively.

The pattern of coactivator contacts is closely reflected in the case of VP16-H1^413–452^. The overall areas contacted overlap extensively with the TAD in either the *cis*- or *trans*-orientation but cover a larger region in the *trans*-orientation (especially in contact area III; Figure 7b,c; Figure S4). Even in terms of the energetics of van der Waals contributions the differences observed are minimal. The detailed computational modelling and results of analyses shown above provide further insights into the types and variety of conformations achievable under physiologically relevant conditions.

## 4. Discussion

Various Mediator (MED) subunits have been identified as potential interaction partners (coactivators) for gene-specific transcription factors (GSTFs), including p53 binding to MED1 and MED17 [41] or MED25 [14], Pdr1 to GAL11/MED15 [42,43], VP16, ETV5, Lana-1 and IE62 to MED25 [13,16,17,20,44,45,46,47,48]. Traditional models envisaged that local interactions between GSTFs and members of the basal transcriptional machinery assembled at the transcription start site provide a relatively confined environment for TADs to bind to coactivators due to the high-density, synergistic binding of transcription factors to enhancers [49], thus allowing even brief contacts (lasting only milliseconds; e.g.) [7] and energetically weak interactions (KD in micromolar range; e.g.) [7,12,13,14,16,50] to become functionally relevant. More detailed investigations focusing on the molecular details of TAD-coactivator interactions identified common principles, such as the induction/stabilization of local α-helical structures and the functional importance of large hydrophobic side chains (for example [7,12,13,14,16,21,39,51,52,53]). Rather gratifyingly, despite the challenges of combining the simulation of intrinsically disordered TADs with more stably structured coactivators within a single model, atomistic molecular dynamics simulations of coactivator TAD complexes generally match the experimental facts well and are thus potentially capable of offering valid insights into questions that are not easily experimentally accessible [14,54,55,56]. Furthermore, computational models not only offer insights into the structural properties of ‘fuzzy complexes’, but also uncover energetic aspects of the highly dynamic binding events that confirm the key functional role of certain hydrophobic residues (Figure 4 and Figure 5) [9]. We were therefore encouraged to investigate the binding mode of TADs to the spatially restricted cleft of MED25-ACID in more detail. MED25-ACID deviates considerably in structural terms from other coactivator domains constructed from α-helix bundles where TADs bind to a superficial groove [7,9,50]. For example, the GAL11-KIX and GAL11-ABD1 domains consist of intertwined helices that create various crevices and pockets suitable for burying the large hydrophobic sidechains in TADs [9,43]. In contrast, the central β-barrel structure of MED25-ACID is dynamically rigid (Figure 1, Figure 6 and Figure 7) and its convex surface is structurally less able to provide distinct binding pockets. Loops and α-helical elements emerging from the β-barrel create a distinct cleft-like structure that has been shown to accommodate various TADs [12,13,14,16]. Binding of a TAD within such a cleft imposes, however, restrictions on the angle of the rod-like α-helical TAD structures within the fuzzy complexes ( typically within 60^o^ (Figure 3c,d; Figure S2)) in comparison to up to 180^o^ rotation accessible on the GAL11-ABD1 domain [9]). Since a switch of TAD orientation on MED25-ACID would require an ability of TADs to rotate by at least 180^o^ once bound, our data suggest that the recruitment of a TAD into the MED25 cleft is likely to be bidirectional, which means that after the initial binding event the approximate orientation of a TAD is fixed in one of two possible directions, which we refer to here as the *cis*- and *trans*-orientations. Once positioned within the coactivator cleft, no change from *cis* to *trans*, or vice versa appears possible without dissociation and reassociation (this statement is supported by the distinct and non-overlapping phase spaces occupied by TADs when bound in opposite orientations, Figure 3c,d). At this stage, the structural aspects of the recruitment of TADs to MED25 (or any other TAD/coactivator system) are not sufficiently well understood. While it is principally possible that TADs are recruited predominantly in a preselected orientation, the great flexibility of the intrinsically disordered domains of GSTFs containing TADs—Combined with the fact that multiple TADs from different GSTFs may compete for coactivator binding sites—Makes this, however, a less likely option. We rather believe that at least a subset of TADs, represented here by ETV5^38–68^ and VP16-H1^413–452^, are capable of binding to MED25-ACID in either orientation with similar predicted affinity (Figure S3). They achieve this by utilizing different functional residues present in their extended transactivation motifs (Figure S1; Figure 4 and Figure 5), as well as by contacting the MED25 cleft in a characteristic manner ( S4, ). We identified four different contact areas (I-IV) within an extended MED25 cleft that are made up from the β3 and β5 strands forming the β-barrel, in conjunction with the small α1 and the longer α3 helices (Figure 7). Interestingly, an additional flexible loop (contact area I; MED25^410–430^) is also involved in TAD binding. Contact area III (MED25^490–510^) is bound in a distinctly localized manner by ETV5^38–68^ in the *cis*-, and by VP16-H1 ^413–452^ in the *trans*-orientation, whereas ETV5^38–68^ in the *trans*-, and VP16-H1^413–452^ in the *cis*-orientation, make contacts over a more extended area, including around ten more amino acids on the N-terminal side (Figure S4). As far as the detailed structural aspects of the TAD motif are concerned, binding in each orientation employs a characteristic, but distinct set of residues (Figure 4 and Figure 5). As was previously shown for the GAL11-ABD1/ GCN4-TAD interaction, the van der Waals contacts formed between TADs and coactivator surfaces are highly dynamic. Some of the interactions are stable throughout the course of a simulation [9] but substituted by other residues in another simulation. Even residues that form unusually stable contacts (such as ETV5-W^57^ (Figure 4a,b) and VP16-F^442^ (Figure 5a,b)) fluctuate in binding strength and are aided by a series of other nearby residues making a series of weaker and more transient contacts. It is notable that the ETV5-W^57^ [20] and VP16-F^442^ residues that were previously identified in mutagenesis screens [21,39], play a dominant role in simulations of the two TADs in the *cis*-orientation, whereas the TADs in the *trans*-orientation are bound by a more diverse and variable set of residues (Figure 4 and Figure 5). We speculate that the fact that ETV5-W^57^ and VP16-F^442^ are so critical for binding to the coactivator in one of the orientations makes them much more vulnerable in mutagenesis screens than the more distributed type of binding observed in the opposite orientation. Such an interpretation of our data also has implications for understanding the primary structure of eukaryotic TADs. Researchers have searched for common motifs that would allow TADs to be identified on the amino acid sequence level and observed some common patterns. One common assumption behind the search for a distinct motif is that it is relatively compact and asymmetric ([38,40,57,58]; 9 amino acid TAD [2,37,57,58]; Figure S1). In light of the observed bidirectional binding ability of both ETV5^38–68^ and VP16-H1^413–452^ TADs employing a wide range of residues and coactivator contact areas in computational simulations, it seems likely that a search for a short TAD consensus motif may be heading in the wrong direction, because TADs may be substantially larger, diverse and adaptable to specific structural environments than previously assumed.

## 5. Conclusions

Recent developments in the field of gene expression have highlighted the unexpected plasticity of molecular recognition events involving intrinsically disordered TADs [59]. We demonstrate that two TADs (ETV5^38–68^ and VP16-H1^413–452^) bind to the same region of the MED25 coactivator and display a pattern of shared and distinct binding modes. Altering the orientation of the TAD with respect to the coactivator binding site led to a change in the dominant residues involved in the interaction but for both TADs binding was maintained in either orientation. This allows for flexibility and redundancy in these interactions.

## Supplementary Materials

The following are available online at https://www.mdpi.com/2218-273X/10/9/1205/s1, Figure S1: Matches within ETV5^38–68^ and VP16-H1^413–452^ TADs to activation consensus motifs, Table S1: Detailed breakdown of secondary structure elements formed within ETV5^38–68^ and VP16-H1^413–452^ TADs after MCMC simulation, Figure S2: Distribution of angles between MED25 and TADs, Figure S3: MM-GBSA results of the total ΔG_Binding_ energy contribution of ETV5^38–68^ binding, Figure S4: MM-GBSA results of the van der Waals energy contributions of MED25 residues.
