# Supplementary material for: Multivalent and Bidirectional Binding of Transcriptional Transactivation Domains to the MED25 Coactivator
Source: Biomolecules. 2020 Aug 19;10(9):1205. doi: 10.3390/biom10091205 (PMC7564715; doi:10.3390/biom10091205)
Supplement: Supplementary file 1 [file biomolecules-10-01205-s001.pdf]

(a)

```
     47       57
DLAHDSEELFQDLSQLQEAWLAEAQVPDDEQ
      EELFQDLSQ
             SQLQEAWLA
                 EAWLAEAQV
```

	9aa TAD Match
EELFQDLSQ	75%
SQLQEAWLA	75%
EAWLAEAQV	83%

(b)

```
                              439   442
APPTDVSLGDELHLDGEDVAMAHADALDDFDLDMLGDGDS
                                    444
                 DVAMAHADA
                       ADALDDFDL
                           DDFDLDMLG
```

	9aa TAD Match
DVAMAHADA	75%
ADALDDFDL	92%
DDFDLDMLG	100%

(c) (d)

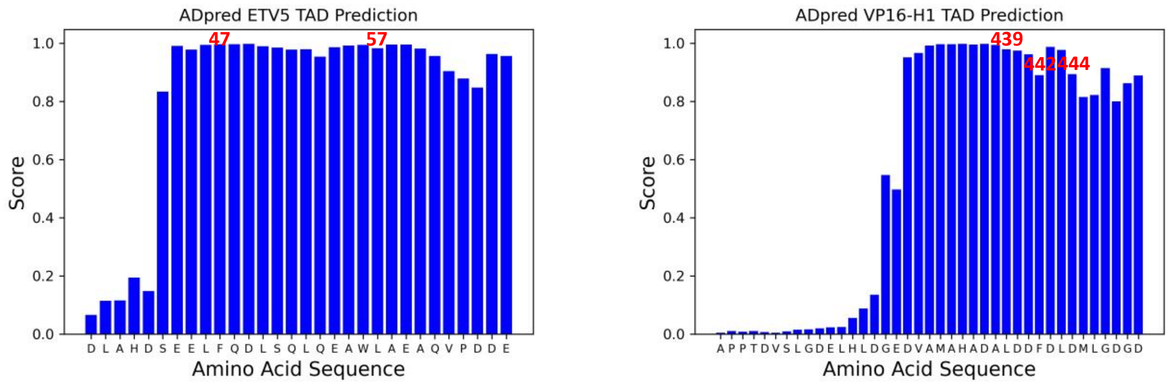

Figure S1. Matches within ETV5$^{38\text{-}68}$ and VP16-H1$^{413\text{-}452}$ TADs to activation domain consensus motifs. (**a**) Matches of ETV5$^{38\text{-}68}$ to the ‘9aa TAD’ consensus. Residues F^{47} and W^{57} are labelled with red numbers. The percentages of the motifs that are matching the ‘low-stringency’ consensus are shown on the right hand side in blue. (**b**) Same as in (a) but for VP16-H1$^{413\text{-}452}$. Residues L^{439}, F^{442} and L^{444} are labelled with red numbers. (**c**) Matches of ETV5$^{38\text{-}68}$ to a TAD consensus motif derived from yeast TADs. The highest score, 1.0, represents a perfect match. (**d**) Same as in (c) but for VP16-H1$^{413\text{-}452}$.
Predictions based on the 9aa TAD prediction tool on https://www.med.muni.cz/9aaTAD/ (for panels [a] and [b]) and https://adpred.fredhutch.org/ (panels [c] and [d]).

Secondary Structure Elements	ETV5$^{38\text{-}68}$	ETV5$^{38\text{-}68}$	VP16-H1$^{413\text{-}451}$	VP16-H1$^{413\text{-}451}$
β-Sheet (para)	0.22%	**β-Sheet: 2.97%**	0.75%	**β-Sheet: 7.33%**
β-Sheet (anti)	2.75%		6.58%	
3_{10} Helix	3.33%	**α-Helix: 44.15%**	5.65%	**α-Helix: 45.89%**
α-Helix	40.78%		40.08%	
π-Helix	0.04%		0.16%	
Turn	9.76%	**Turn/Bend: 18.41%**	19.59%	**Turn/Bend: 32.42%**
Bend	8.65%		12.83%	

Table S1. Detailed breakdown of secondary structure elements formed within ETV5$^{38\text{-}68}$ and VP16-H1$^{413\text{-}452}$ TADs after MCMC simulation. Both ETV5$^{38\text{-}68}$ and VP16-H1$^{413\text{-}452}$ TADs contain overall a similar proportion of α-helices that are formed transiently in absence of binding to a coactivator target, but VP16-H1$^{413\text{-}452}$ contains a higher proportion of disorder-generating turns and bends.

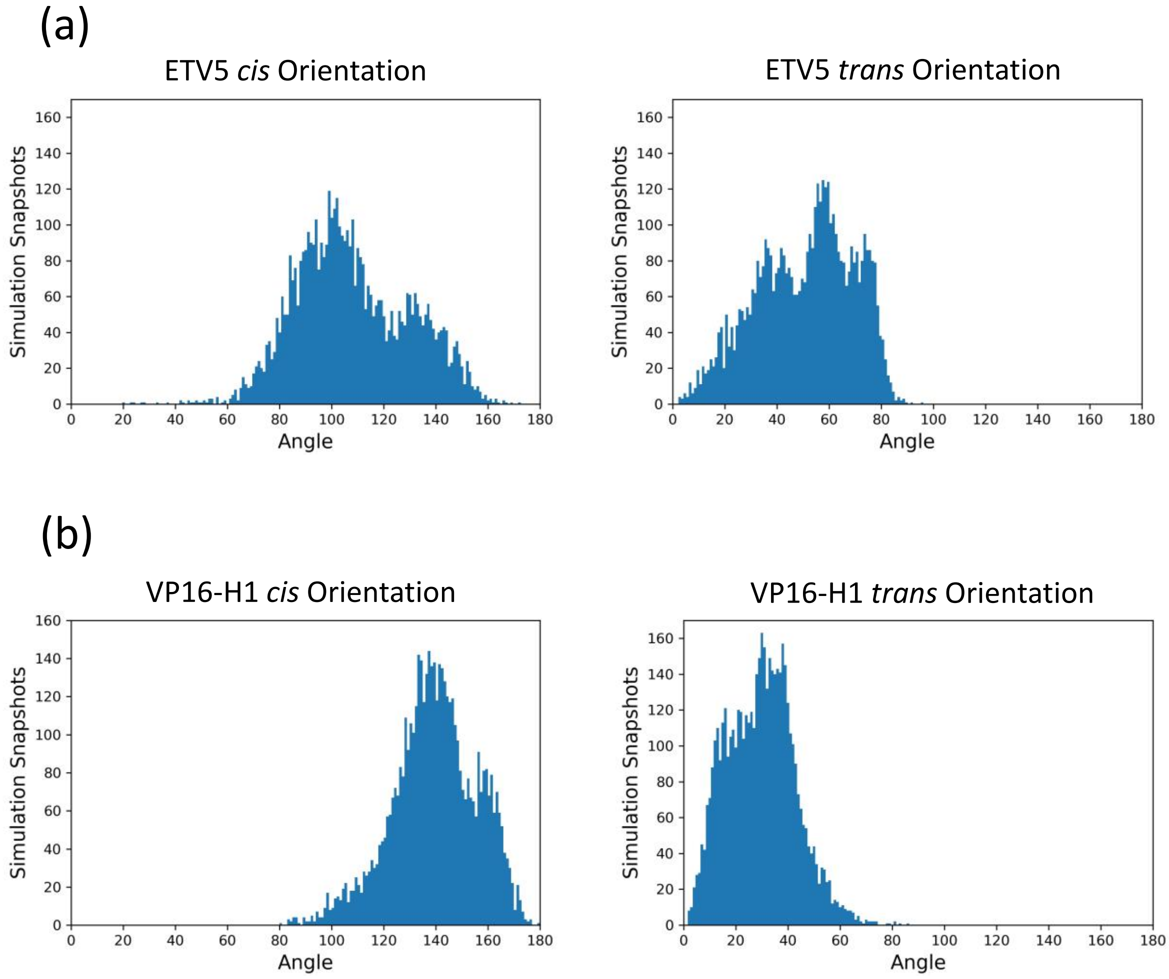

Figure S2. Distribution of angles between MED25 and TADs. (**a**) Distributions of angles between the α-helical portion of ETV5$^{38\text{-}68}$ and the direction of the β3 strand of MED25 in *cis*- and *trans*-orientations (left and right panel, respectively). (**b**) Same as in (a) but for VP16-H1$^{413\text{-}452}$.

(a) (b)

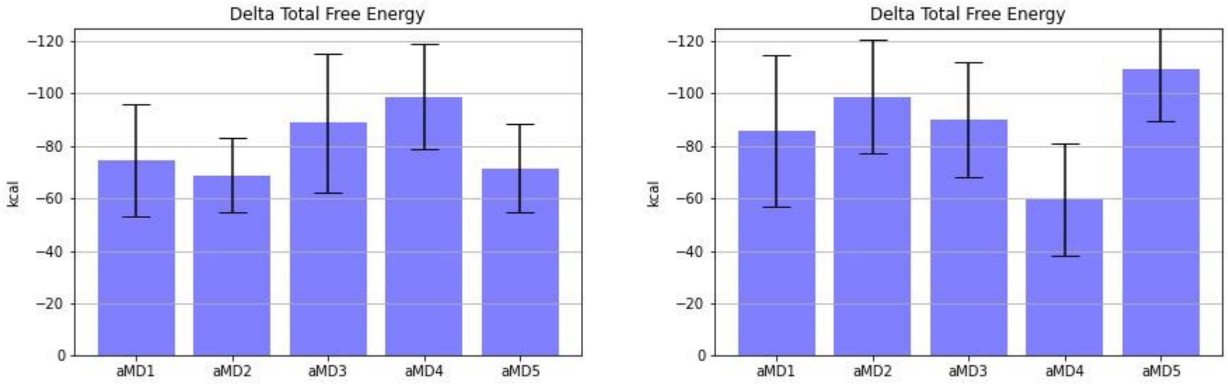

(c)

	Average	Standard Deviation
**aMD1 *cis***	-74.4	21.5
**aMD2 *cis***	-68.9	14.3
**aMD3 *cis***	-88.8	26.5
**aMD4 *cis***	-98.7	20.1
**aMD5 *cis***	-71.4	16.8

	Average	Standard Deviation
**aMD1 *trans***	-85.8	28.9
**aMD2 *trans***	-98.8	21.8
**aMD3 *trans***	-89.0	21.9
**aMD4 *trans***	-59.6	21.3
**aMD5 *trans***	-109.5	19.9

Figure S3. MM-GBSA results of the total $\Delta G_{Binding}$ energy contributions of $ETV5^{38\text{-}68}$ binding in (**a**) the *cis*-orientation, or (**b**) the *trans*-orientation. $\Delta G_{Binding}$ combines van der Waals and electrostatic interactions. (**c**) Table showing the values as numerical results. The data is averaged from five independent aMD simulations/panel (labelled on the x-axis) and the error bars show the standard deviation.

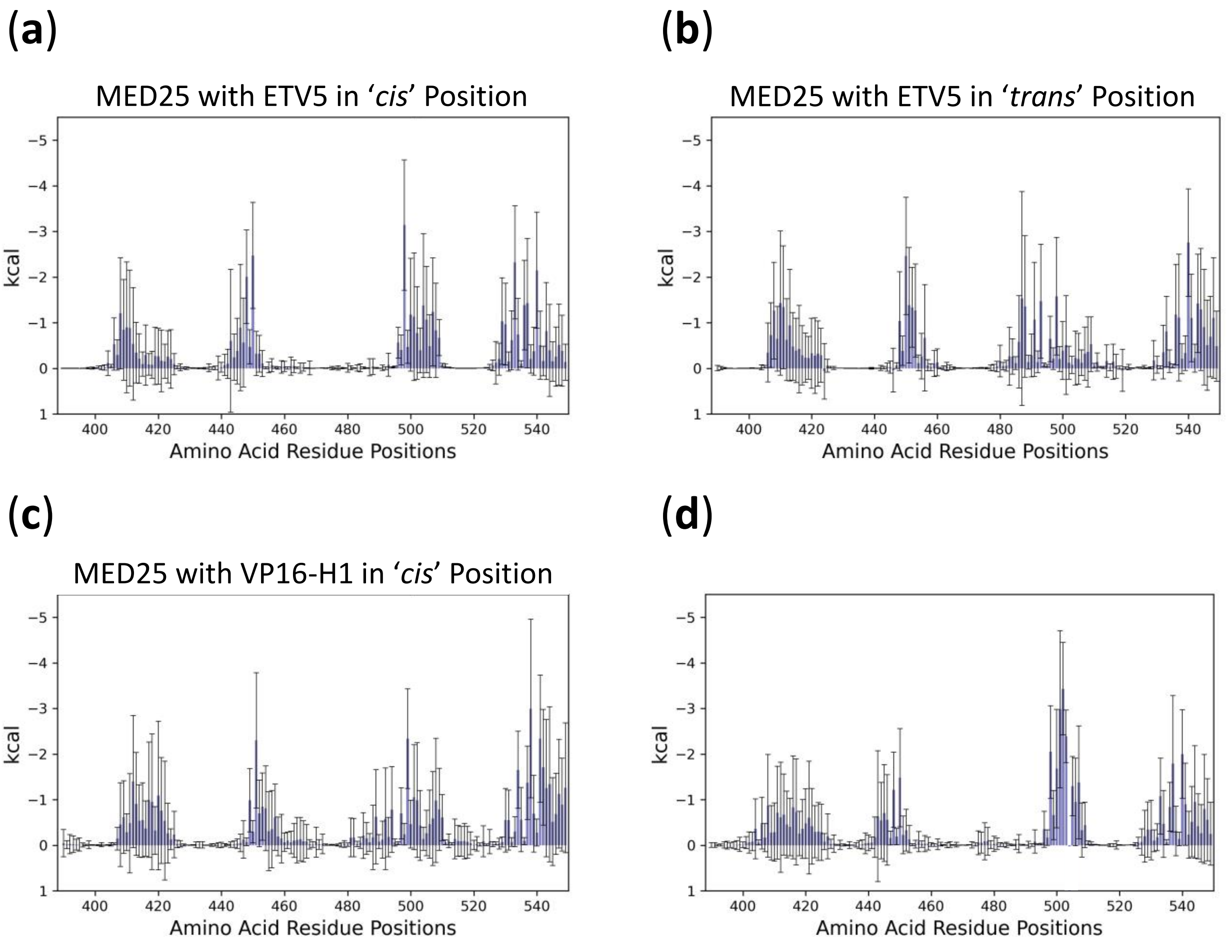

Figure S4. MM-GBSA results of the van der Waals energy contributions of MED25 residues. Each data set shows the average value for each residue from five aMD simulations and the standard deviation as error bars. (**a**) Residue-specific energies during binding of $ETV5^{38-68}$ in the *cis*-orientation. (**b**) Residue-specific energies during binding of $ETV5^{38-68}$ in the *trans*-orientation. (**c**) Same as in (a) but for VP16-$H1^{413-452}$. (**d**) Same as in (b) but for VP16-$H1^{413-452}$.
